# New insights into keloid pathogenesis: biomarker potential for CDK7 and DDB2

**DOI:** 10.3389/fcell.2025.1718189

**Published:** 2025-11-19

**Authors:** Weiqiang Zhang, Fujun Wang, Yixun Zhang, Lusheng Xu, Lujia Mao, Xiaoxiang Wang, Ronghua Yang

**Affiliations:** 1 The First Clinical School of Medicine, Guangdong Medical University, Zhanjiang, Guangdong, China; 2 School of Basic Medicine, Qiqihar Medical University, Qiqihar, Heilongjiang, China; 3 Department of Burn and Plastic Surgery, Guangzhou First People’s Hospital, Guangzhou, Guangdong, China; 4 Plastic Surgery Department, Foshan First People’s Hospital, Foshan, Guangdong, China; 5 Department of Burn Surgery, The First Affiliated Hospital of Sun Yat-Sen University, Guangzhou, Guangdong, China

**Keywords:** keloid, GEO, CDK7, DDB2, bioinformatics

## Abstract

**Introduction:**

Keloid formation is a prevalent dermatological condition characterized by abnormal dermal connective tissue proliferation. Despite ongoing research, the underlying mechanisms of keloid formation remain insufficiently understood. The aim of this research is to identify and verify molecular biomarkers associated with keloid and to explore potential therapeutic targets.

**Methods:**

Transcriptomic data from keloid tissue specimens and normal skin controls were retrieved from the Gene Expression Omnibus (GEO) database. We performed differential expression and functional enrichment analyses after batch effect correction. We performed differential gene analysis, weighted Gene Co-expression Network Analysis (WGCNA), and protein-protein interaction (PPI) analyses to verify hub genes, explore their functions, and evaluate their connection to keloid formation, therapeutic potential, and immune-related characteristics. Key genes were validated through experimental assays.

**Results:**

679 differentially expressed genes (DEGs) were identified. Through WGCNA and Venn diagram analysis, 41 DEGs most closely associated with keloid were identified. These 41 overlapping DEGs were confirmed to be markedly involved in metabolic pathways, nucleotide excision repair, and amino acid biosynthesis by functional enrichment analysis. PPI analysis identified CDK7 and DDB2 as hub genes, each demonstrating strong diagnostic performance in ROC curve analysis (AUC = 0.80), with comparable results in validation datasets (AUC = 0.86). Basic experiments confirmed higher expression of CDK7 and DDB2 in keloid tissue compared to normal skin.

**Conclusion:**

Our findings demonstrate that CDK7 and DDB2 are promising biomarkers for diagnostic and potential therapeutic targets in keloid, providing novel insights into its pathogenesis and offering promising druggable targets.

## 1 Introduction

Keloid, a frequent skin disorder, originates from prolonged dysregulated wound healing. In this condition, the equilibrium between collagen production and breakdown is disrupted by a combination of external triggers and internal predispositions (Pauline et al., 2009). Although benign, keloids exhibit growth patterns similar to neoplasms, with aggressive expansion, invasion into surrounding normal tissue, and a tendency to protrude above the skin surface beyond the original wound site. They often present with a hard texture, crab-like appearance, and symptoms like itching or pain, impacting aesthetics and, in severe cases, impairing skin and joint function (Silvian et al., 2019).

Recent reviews on keloid pathogenesis highlight two primary theories: the inflammatory theory and the tumor theory. The central immunocytes implicated in keloid pathogenesis comprise macrophages, lymphocytes, mast cells, and neutrophils, with earlier studies noting increased infiltration of macrophage populations, T-cells, and mast cells clusters within keloid lesions compared to normal dermal tissue (Gauglitz et al., 2011; Shaker et al., 2011). Keloid tissue exhibit significant upregulation of multiple pro-inflammatory mediators, including interleukin-6 (IL-6), IL-8, IL-18, and chemokine-like factor-1 (CKLF-1), compared to normal dermal tissue. Moreover, the peripheral blood IL-8 levels in keloid patients are found to be seven times higher than those in healthy individuals (Abdou et al., 2014; Zhang et al., 2016; Tanaka et al., 2019).

In line with the tumor theory, several studies report overexpression and secretome emission of growth factors such as TGF-β, IGF-I, PDGF, and EGF in keloid tissue, with keloid cells demonstrating heightened sensitivity to these factors compared to normal cells (Haisa et al., 1994; Younai et al., 1994; Ohtsuru et al., 2000). Additionally, early keloid studies identified abnormal expression of apoptosis-related genes, including ASY, PEA 15, AVEN, and ADAM12 (Satish et al., 2006; Seifert et al., 2008), alongside inactivation of the tumor suppressor gene P53 (De Felice et al., 2004). Compared to normal skin, keloids exhibit an enhanced capacity for angiogenesis (Yoo and Kim, 2014; Jumper et al., 2015), further supporting their tumor-like behavior and providing a theoretical basis for their unchecked proliferation.

Significant progress has been made in keloid research globally; however, the precise pathogenesis of keloid remains unclear, necessitating further extensive research. This lack of clarity complicates efforts to achieve effective clinical outcomes. Current treatments include corticosteroid patches (Yoshino et al., 2018), hypocrellin (Wu et al., 2024), silicone gel sheets (O'Brien and Jones, 2013), corticosteroid injections (Zhuang et al., 2021), 5-fluorouracil injections (Hietanen et al., 2019; Zhang et al., 2025), cryotherapy (O’Boyle et al., 2017), and surgery (Mustoe et al., 2002), with excision being the most direct method. Despite these options, recurrence remains common, imposing a psychological burden on patients. Consequently, there is a critical need for deeper investigation into keloid pathogenesis and prevention. Identifying novel keloid markers and elucidating their mechanisms are key steps forward.

**FIGURE 1:**
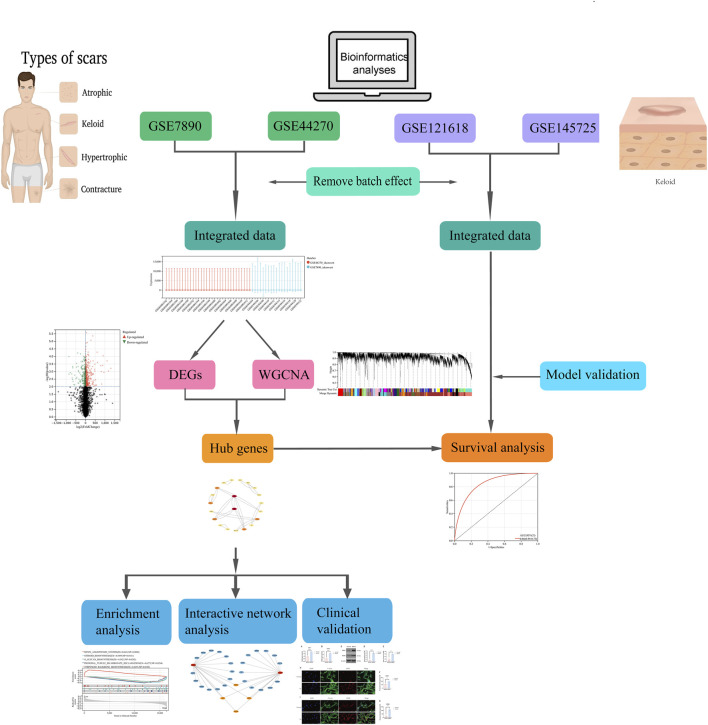
Research framework and methodological process.

Bioinformatics has proven invaluable for studying keloid formation mechanisms and can be used to identify pathogenesis-related biomarkers, opening pathways for new diagnostic strategies (Yin et al., 2022; Shixin et al., 2024). This study analyzed gene expression profiles from the GEO database, utilizing bioinformatics methodologies to assess gene expression associated with keloid, perform functional analyses, construct PPI networks, and examine immune cell infiltration within the keloid tissue environment. Ultimately, we identified keloid - associated biomarkers, explored their roles in pathogenesis, and provided novel insights into potential therapeutic strategies. The overall flow chart for the research design is depicted in Figure 1.

## 2 Materials and methods

### 2.1 Data collection

Four gene expression datasets from keloid patients were obtained from the GEO database. Precisely, dataset GSE44270 includes 18 keloid and 14 control samples, GSE7890 includes 10 keloid and 9 control samples, GSE145725 includes 9 keloid and 10 control samples, and GSE121618 includes 5 keloid and 6 control samples. Datasets GSE44270 and GSE7890 were sequenced on GPL570, while GSE145725 and GSE121618 were sequenced on GPL16043. An integrated dataset was generated using Empirical Bayes methods (Johnson et al., 2007) to remove batch effects, and the batch-effect-free matrix was visualized with UMAP plots. The R package inSilicoMerging (Taminau et al., 2012) was then used to merge datasets GSE44270 with GSE7890 and GSE121618 with GSE145725.

### 2.2 Identification and analysis of keloid-associated differentially expressed genes

To authenticate DEGs across keloid and normal specimens within the integrated dataset and evaluate their pathological contributions, we performed the R package limma (Ritchie et al., 2015) for differential expression analysis. Genes were deemed upregulated if they had a log_2_FC > 0 and P < 0.01, whereas those with log_2_FC < 0 and P < 0.01 were seen as downregulated. Volcano plots and heatmaps were generated to visualize the DEGs and their clustering patterns.

### 2.3 Functional enrichment analysis

Standardized terms for describing gene functions are provided by Gene Ontology (GO), which covers biological processes (BP), molecular functions (MF), and cellular components (CC). The Kyoto Encyclopedia of Genes and Genomes (KEGG) delivers comprehensive data regarding genes and metabolic pathways. Functional enrichment profiling of DEGs was conducted through GO and KEGG pathway analyses to elucidate statistically significant pathways, with analytical outcomes graphically represented via bubble plot visualizations (statistical threshold *p* < 0.05).

We employed Gene Set Enrichment Analysis (GSEA) to detect significantly enriched gene sets under specific conditions. Gene sets were sourced from the Molecular Signatures Database (MSigDB), and GSEA was performed using FDR < 0.25 and *p* < 0.05 as thresholds. Computational workflows were executed in the R statistical environment via the clusterProfiler toolkit for comprehensive enrichment profiling (Yu et al., 2012; Wu et al., 2021).

### 2.4 Weighted gene co-expression network analysis (WGCNA)

A scale-free co-expression network architecture was constructed using the WGCNA R package, enabling systematic investigation of transcriptomic interconnectivity patterns and functional genotype-phenotype interrelationships through topological analysis (Langfelder and Horvath, 2008). We selected an optimal soft threshold and enabled the construction of a scale-free co-expression network to validate network modules and hub genes linked to keloid development and progression. Transcriptomic profiles exhibiting correlated expression dynamics were algorithmically partitioned into functional co-expression modules through implementation of dynamic tree-cutting algorithms. Module eigengenes (MEs) were computed to facilitate the merging of modules with a distance of under 0.25. Each module’s ME, representing its gene expression profile, was used to analyze module-phenotype associations. Module membership (MM) values reflect the relationship between a gene’s expression and its module’s ME, showing how strongly the gene is linked to the module. Gene significance (GS) is the correlation between a gene’s expression and the phenotype, reflecting the gene’s relevance to the phenotype.

### 2.5 Identification and analysis of hub genes and PPI network

To validate key genes linked to keloid, we performed the R package VennDiagram to find the intersection of DEGs and WGCNA-identified genes, followed by GO and KEGG pathway enrichment analyses. We utilized the STRING database (Szklarczyk et al., 2019) to identify known proteins and forecast interactions. We loaded the overlapping genes from the Venn diagram into STRING to construct a PPI network. Then we used Cytoscape software (Smoot et al., 2011). to visualize the PPI network. Based on expression differences, enrichment functions, and PPI network analyses, the degree values of all genes were calculated, with those having higher values deemed as hub genes. Box plots were then utilized to visualize differential expression of the key genes in keloid and matched normal dermal tissues.

### 2.6 Receiver operating characteristic (ROC) curve for hub genes

Based on gene expression levels and patient survival times, we utilized the survival R package to construct ROC curves. Gene expression levels were evaluated for their diagnostic value in patient survival prediction using the area under the curve (AUC). The prognostic predictive capacity of key gene was evaluated through Cox proportional hazards regression modeling implemented in the survival R package. The pROC package’s CI function was applied to calculate AUC and confidence bounds. Additionally, Kaplan-Meier (KM) curves and prognostic heatmaps were generated to assess the correlation of key genes with keloid.

### 2.7 Analysis of hub genes’ drug activity, transcription factor, and microRNA interaction networks

miRNAs associated with CDK7 and DDB2 were identified by the miRWalk database (http://mirwalk.umm.uni-heidelberg.de/), selecting the top 10 miRNAs based on binding potential and energy criteria. Transcription factors (TFs) for CDK7 and DDB2 were predicted using the ChEA3 database (https://maayanlab.cloud/ChEA3/), with the top 30 TFs selected for visualization based on scoring criteria. Therapeutic targets associated with central regulatory genes were systematically mined from the Drug-Gene Interaction Database (DGIdb, https://www.dgidb.org), followed by computational mapping and topological visualization of pharmacological interaction networks using Cytoscape’s analytical platform.

### 2.8 Immune infiltration analysis

The immune microenvironment is composed of fibroblasts, mesenchymal cells, immune cells, and inflammatory cells, as well as various cytokines and chemokines. Assessing immune cell infiltration is critical for determining the predictive value of disease progression and therapeutic outcomes. We used CIBERSORT to evaluate immune cell infiltration within the microenvironment and to systematically investigate immune cells interactions with key genes. Complementary analysis of datasets (GSE44270/GSE7890) was performed to resolve 22 distinct immune cell type proportions across biological replicates.

### 2.9 Cell culture

In this study, keloid tissue was sourced from Guangzhou First People’s Hospital. Its acquisition followed the Declaration of Helsinki and was authorized by the hospital’s Medical Ethics Committee. Fibroblasts isolated from keloid patient tissue were cultured and expanded, with passage 5 cells used in experiments. The cells were maintained in Dulbecco’s Modified Eagle Medium (DMEM; Gibco, United States) containing 10% fetal bovine serum (Gibco, United States) and antibiotics (Servicebio, China). Cultures were incubated at 37 °C in a 5% CO_2_ atmosphere, with the medium replaced every 48 h, and cells subcultured upon reaching 70%–80% confluence.

### 2.10 Quantitative real-time polymerase chain reaction (qRT-PCR)

TRIzol was used to extract total RNA, after which cDNA was synthesized using the RevertAid Reverse Transcriptase kit (Thermo Fisher Scientific, United States). With actin as the internal control, we performed quantitative PCR on the synthesized cDNA through SYBR Green RT-PCR (Takara Biotechnology Co., Ltd., Japan). Then the 2^−△△CT^ method was applied to calculate relative gene expression levels (Yu et al., 2024). Primer sequences are provided in Table 1.

**TABLE 1:** Basic information of qRT-PCR primers.

Gene	Forward primer (5′ to 3′)	Reverse primer (5′ to 3′)
ACTIN	GTCCACCGCAAATGCTTCTA	TGCTGTCACCTTCACCGTTC
CDK7	AAGTGCACCTCTTTGCCCAA	GGTCCTCGTAAGGACTCGAT
DDB2	AGGACTACATGACCCTGCGA	CCTTCTTCCAGTGCATGCTG

### 2.11 Western blotting

Protein extraction from HaCaT cells was conducted using a radioimmunoprecipitation assay (RIPA) buffer with a protease inhibitor cocktail (Beyotime, China). Protein quantification was performed using a BCA assay system (Thermo Fisher Scientific), followed by electrophoretic separation of 20 μg protein lysates on 10% SDS-PAGE. Resolved proteins were electrophoretically transferred to PVDF membranes, blocked with 5% BSA, and probed with primary antibodies (4 °C, 16 h). Subsequent incubation of secondary antibodies (room temperature, 1 h) preceded chemiluminescent detection using an AI800 imaging platform (GE, United States). Quantitative densitometric analysis was conducted with ImageJ. Antibodies were sourced as follows: CDK7 (67889-1-Ig, 1:1000, Proteintech Group), DDB2 (10431-1-AP, 1:1000, Proteintech Group), and β-Actin (TDYO51, 1:5000, TDY BIOTECH).

### 2.12 Immunofluorescence assay

Immunofluorescence protocols commenced with cellular immobilization using 4% paraformaldehyde (30 min), followed by triple rinsing in PBS and membrane permeation with 0.1% Triton X-100/TBST solution (room temperature, 30 min). Non-specific binding sites were neutralized with 5% BSA blocking buffer (30 min). Primary antibody incubation (16 h, 4 °C) preceded application of secondary antibodies linked to horseradish peroxidase (goat anti-rabbit, Affinity, 1:500; room temperature, 1 h) for signal amplification. DAPI was used to stain nuclei for 10 min (Servicebio, China). Imaging was conducted with a confocal microscope (Zeiss, Germany).

### 2.13 Statistical analysis

All data processing and analyses were implemented within the R statistical environment (v4.0.2), employing Student's t-test for parametric inter-group comparisons with a predefined significance threshold (*p* < 0.05) across all analyses.

## 3 Result

### 3.1 Expression and analysis of differentially expressed genes

**FIGURE 2:**
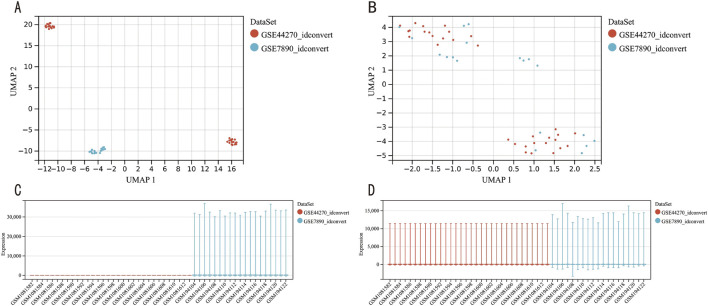
GEO data batch correction. **(A)** UMAP plot of the dataset before batch correction. **(B)** UMAP plot of the dataset after batch correction. **(C)** Gene expression level analysis of the dataset prior to batch correction. **(D)** Gene expression level analysis of the dataset following batch correction.

**FIGURE 3:**
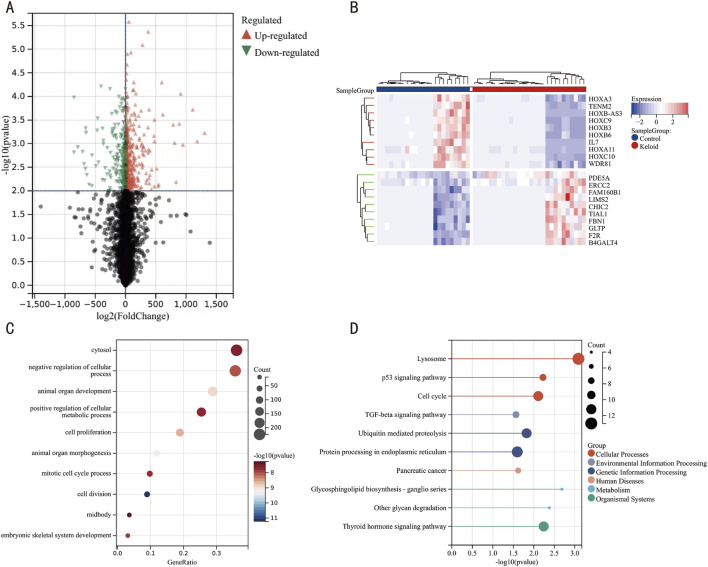
Heatmap, Volcano Plot, GO, and KEGG Pathway Analyses of DEGs. **(A)** Volcano plot of DEGs, with orange nodes indicating upregulated and green nodes indicating downregulated DEGs. **(B)** Heatmap of DEG expression levels, where crimson columns represent keloid specimens and azure columns represent control matched control cohorts. **(C)** Functional enrichment landscape visualization of GO terms, with bubble size correlating with gene ratio and chromatic intensity reflecting statistical significance [-log10 (P-value)]. **(D)** Pathway interaction network analysis through KEGG mapping, highlighting enriched biological cascades through scaled circular nodes.

We first removed the batch effects from datasets GSE7890 and GSE44270 (Figure 2), resulting in an integrated dataset comprising 28 keloid samples and 23 control samples. Differential expression analysis on this integrated dataset identified 679 DEGs, including 428 upregulated and 251 downregulated genes, as visualized in a volcano plot (Figure 3A). A heatmap of the top 20 DEGs (Figure 3B) highlights distinct expression patterns between keloid and control samples.

To delineate the molecular mechanisms underlying keloid formation, we implemented multidimensional functional profiling through GO annotation and KEGG pathway topology mapping of DEGs from the integrated data. GO enrichment indicated significant associations with biological processes (BP) such as system development, suppression of cellular processes, and enhancement of metabolic processes; cellular components (CC) like the cytoplasm; and molecular functions (MF) such as catalytic activity (Figure 3C, details in Table 2). KEGG pathway analysis revealed enrichments primarily related to lysosomes, cell cycle regulation, ubiquitin-mediated protein degradation, and protein processing in the endoplasmic reticulum (Figure 3D, details in Table 3). These findings suggest that keloid formation may be driven by disruptions in cellular proliferation, metabolic regulation, and key processes such as lysosomal degradation, protein processing, and cell cycle regulation. These abnormalities likely contribute to excessive fibrous tissue accumulation and atypical wound healing, which may be central to keloid pathogenesis.

**TABLE 2:** GO enrichment analysis results.

Ontology	ID	Description	GeneRatio	BgRatio	pvalue	p.adjust	qvalue
BP	GO:0048731	System development	220/614	4,670/17,910	3.63E-08	2.20E-05	1.84E-05
BP	GO:0048523	Negative regulation of cellular process	219/614	4,580/17,910	1.09E-08	1.34E-05	1.12E-05
BP	GO:0009893	Positive regulation of metabolic process	167/614	3,313/17,910	4.66E-08	2.20E-05	1.84E-05
CC	GO:0005829	Cytosol	224/621	4,909/18,675	3.04E-08	1.99E-05	1.61E-05
MF	GO:0003824	Catalytic activity	208/604	4,828/16,967	0.000642816	0.086443093	0.078550867

**TABLE 3:** KEGG enrichment analysis results.

Ontology	ID	Description	GeneRatio	BgRatio	pvalue	p.adjust	qvalue
KEGG	hsa04142	Lysosome	13/303	123/7,914	0.000816951	0.232831095	0.225306544
KEGG	hsa04110	Cell cycle	11/303	124/7,914	0.007793219	0.370177916	0.358214641
KEGG	hsa04120	Ubiquitin mediated proteolysis	11/303	136/7,914	0.015005016	0.584453081	0.565564939
KEGG	hsa04141	Protein processing in endoplasmic reticulum	12/303	166/7,914	0.025338959	0.584453081	0.565564939

### 3.2 WGCNA

**FIGURE 4:**
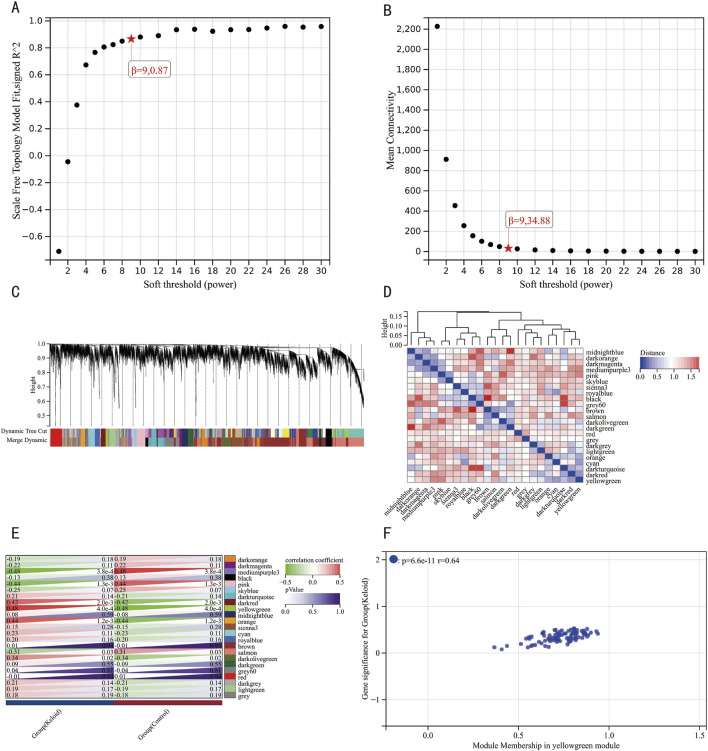
WGCNA Results. **(A)** Scale-free network topology fitness metrics across discrete soft-thresholding powers. **(B)** Scale-free network topology fitness metrics across discrete soft-thresholding powers. **(C)** Hierarchical clustering dendrogram of co-expressed gene ensembles. **(D)** Eigengene-based module clustering architecture with topological annotations. **(E)** Module-clinical trait interaction mapping, visualizing synergistic associations (red) and antagonistic relationships (green). **(F)** Gene significance-module membership interconnectivity profiling within the pivotal yellow-green functional module.

We analyzed gene expression profiles from 51 samples in the integrated dataset using WGCNA. We achieved scale independence at 0.87 and an average connectivity of 34.88 after the soft-thresholding power set to 9 (Figures 4A,B). A clustering dendrogram was then constructed, followed by dynamic tree cutting and merging (Figure 4C), resulting in 23 distinct co-expression modules. A module eigengene cluster diagram was also generated (Figure 4D). The correlation analysis between each module and clinical characteristics revealed the yellow-green module demonstrated the most robust synergistic interplay with keloid pathogenesis, while the medium-purple3 cluster displayed pronounced inverse regulatory relationships (Figure 4E). Further MM and GS analyses of the yellow-green module indicated a high correlation with both the module and the keloid phenotype (*r* = 0.64, *p* = 6.6e-11) (Figure 4F).

### 3.3 Identification and analysis of key genes

**FIGURE 5:**
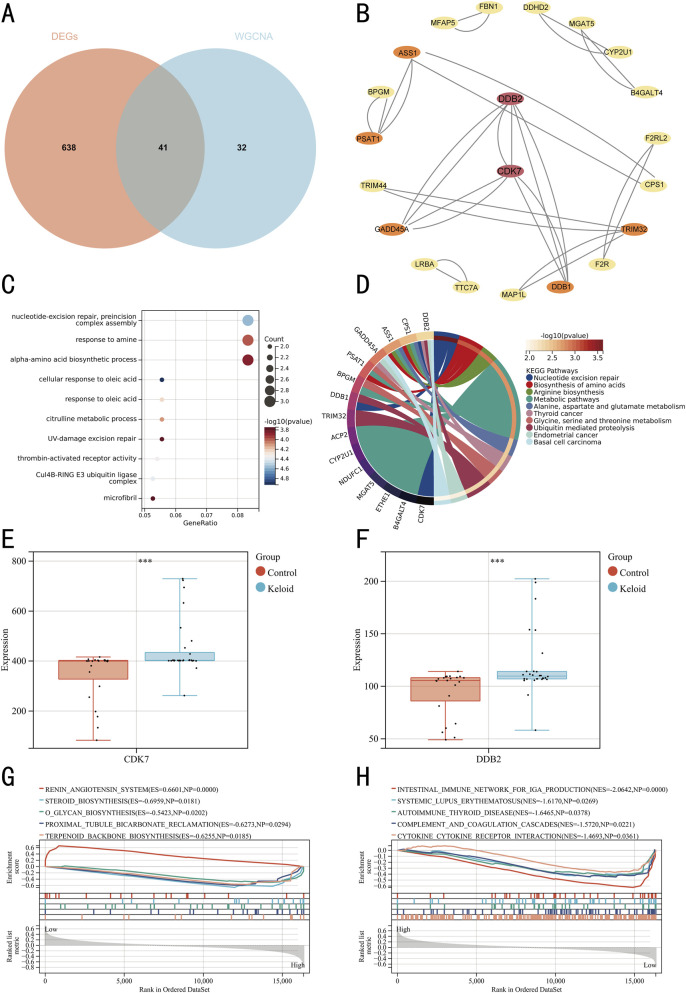
Identification and GSEA of hub genes. **(A)** Venn diagram with two colors representing distinct data sources. **(B)** PPI network of hub genes, with crimson nodes indicating hub genes. **(C)** Bubble plot of GO pathway enrichment analysis for the 41 DEGs. **(D)** Chord diagram of KEGG pathway enrichment analysis for the 41 DEGs. **(E)** Box plot of CDK7 expression levels. **(F)** Box plot of DDB2 expression levels. **(G)** GSEA for hub gene CDK7. **(H)** GSEA for hub gene DDB2.

We extracted 679 DEGs and 73 genes from the yellow-green module, using a Venn diagram to identify an intersection of 41 genes (Figure 5A). GO (Figure 5C) and KEGG (Figure 5D) enrichment analyses of these 41 genes indicated significant enrichment in metabolic pathways, nucleotide excision repair, and amino acid biosynthesis processes. Systematic interrogation of 41 candidate genes through the STRING platform established a PPI network topology containing 21 nodal proteins interconnected via 31 functional edges (Figure 5B). Based on degree scores, CDK7 and DDB2 were identified as key genes, with their expression levels in keloid and control groups visualized via box plots (Figures 5E,F). Based on CDK7 and DBB2, GSEA identified 186 KEGG pathways, with the top 5 pathways for CDK7 being renin angiotensin system, steroid biosynthesis, o-glycan biosynthesis, proximal tubule bicarbonate reclamation, and terpenoid backbone biosynthesis (Figure 5G). For DDB2, the top 5 pathways were intestinal immune network for iga production, systemic lupus erythematosus, autoimmune thyroid disease, complement and coagulation cascades, and cytokine-cytokine receptor interaction (Figure 5H). Detailed pathway information is provided in Tables 4, 5.

**TABLE 4:** GSEA enrichment analysis results of CDK7.

Term	ES	NES	pvalue	FDR	FWER
RENIN_ANGIOTENSIN_SYSTEM	0.6601	1.7358	0	0.1468	0.176
STEROID_BIOSYNTHESIS	−0.6959	−1.5774	0.0181	0.2037	0.592
O_GLYCAN_BIOSYNTHESIS	−0.5423	−1.5978	0.0202	0.2124	0.536
PROXIMAL_TUBULE_BICARBONATE_RECLAMATION	−0.6273	−1.5613	0.0294	0.2127	0.639
TERPENOID_BACKBONE_BIOSYNTHESIS	−0.6255	−1.5815	0.0185	0.219	0.577

**TABLE 5:** GSEA enrichment analysis results of DDB2.

Term	ES	NES	pvalue	FDR	FWER
INTESTINAL_IMMUNE_NETWORK_FOR_IGA_PRODUCTION	−0.6693	−2.0642	0	0	0
SYSTEMIC_LUPUS_ERYTHEMATOSUS	−0.4655	−1.617	0.0269	0.2403	0.471
AUTOIMMUNE_THYROID_DISEASE	−0.4811	−1.6465	0.0378	0.2473	0.376
COMPLEMENT_AND_COAGULATION_CASCADES	−0.4656	−1.572	0.0221	0.2837	0.598
CYTOKINE_CYTOKINE_RECEPTOR_INTERACTION	−0.3752	−1.4693	0.0361	0.3261	0.837

### 3.4 ROC curve analysis of key genes

**FIGURE 6:**
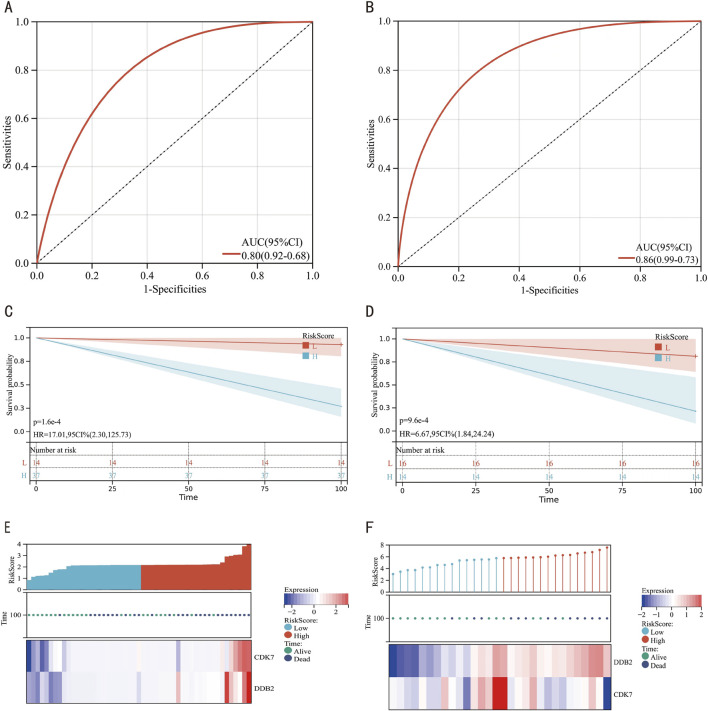
Diagnostic Evaluation of Hub Genes via ROC Curve Analysis. **(A)** ROC curves for hub genes. **(B)** ROC curves for hub genes in the combined datasets GSE121618 and GSE145725. **(C)** KM curve for hub genes. **(D)** KM curve for hub genes in the integrated datasets GSE121618 and GSE145725. **(E)** Prognostic heatmap for hub genes in the combined datasets GSE121618 and GSE145725. **(F)** Prognostic heatmap for hub genes in the combined datasets GSE121618 and GSE145725.

ROC analytical frameworks were established to evaluate the diagnostic potential of CDK7 and DDB2 as keloid biomarker candidates. As shown in Figure 6A, both genes demonstrated strong diagnostic performance (AUC = 0.80). Further ROC validation analysis in datasets GSE121618 and GSE145725 (Figure 6B) confirmed high diagnostic performance (AUC = 0.86). Additionally, KM curves, prognostic heatmaps (Figures 6C,D), and their respective validation datasets (Figures 6E,F) consistently showed a strong association between these key genes and keloid.

### 3.5 Construction of drug activity and transcription factor regulatory networks for hub genes

**FIGURE 7:**
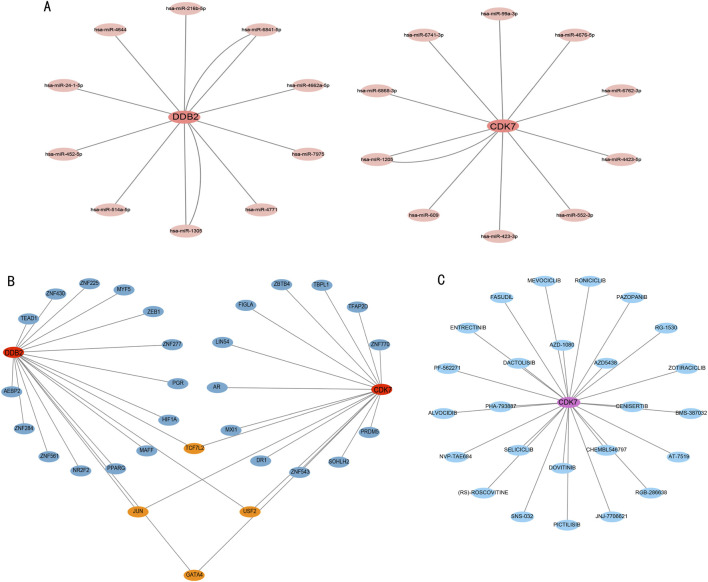
Interactive network analysis. **(A)** DDB2–miRNA and CDK7–miRNA networks. **(B)** DDB2–transcription factor and CDK7–transcription factor networks. **(C)** CDK7–drug interaction network.

The DEG-miRNA network analysis shows that DDB2 and CDK7 are associated with distinct miRNAs (Figure 7A). The transcription factor-target gene interaction network for DDB2 and CDK7 indicates that they share common transcription factors (Figure 7B). Additionally, the drug-target gene interaction network (Figure 7C) suggests that CDK7 is associated with drugs, including entrectinib and alvocidib (both CDK inhibitors), RG-1530, roniciclib, and pazopanib.

### 3.6 Immune infiltration and immune-related factors

**FIGURE 8:**
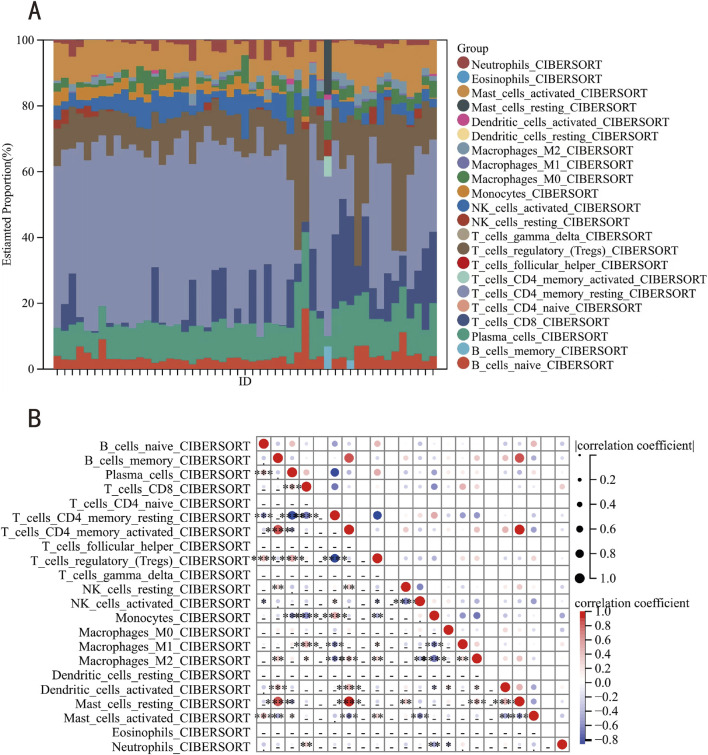
Immune infiltration analysis. **(A)** The relative percentage of 22 immune cells in each sample. **(B)** Relevant bubble diagram of 22 immune cells.

This investigation employed the CIBERSORT computational framework to quantify 22 immune cell type infiltrations across 28 keloid lesions and 23 matched healthy cutaneous specimens (Figure 8A). Complementarily, we conducted multidimensional profiling to delineate the functional interplay between key genes and the various immune cell types. Results indicated that resting mast cells, activated memory CD4^+^ T cells, and memory B cells, among others, showed strong correlations with the key genes, whereas follicular helper T cells, naive CD4^+^ T cells, γ-δ T cells, resting dendritic cells, and eosinophils exhibited no correlation (Figure 8B).

### 3.7 Experimental validation

**FIGURE 9:**
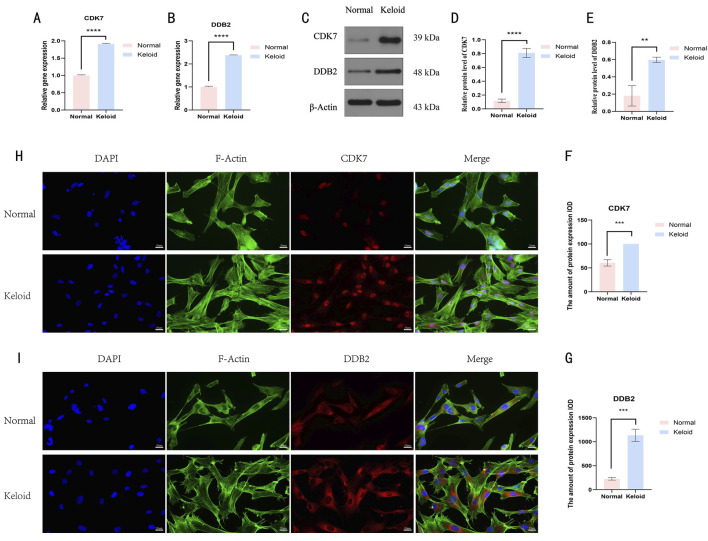
The result of the basic experiment of CDK7 and DDB2. **(A,B)** The plots showed the results of qRT-PCR. **(C–E)** The result of the expression levels of CDK7 and DDB2 proteins in normal skin and keloid. **(F,G)** The result of the total expression levels of CDK7 and DDB2 proteins in normal skin and keloid. **(H,I)** Observation of intracellular localization and expression patterns of CDK7 and DDB2 proteins in normal skin and keloid.The results are presented as mean ± SD. **, ***, **** respectively represent P values of t-test < 0.01, < 0.001, < 0.0001.

Finally, we validated our bioinformatics findings by assessing CDK7 and DDB2 expression levels in tissues from six keloid patients and six normal subjects using qRT-PCR, western blotting, and immunofluorescence. These experiments verified that CDK7 and DDB2 expression in keloid tissue cells was markedly elevated compared to normal tissues (Figure 9), further supporting the reliability and research value of our analysis.

## 4 Discussion

Keloid pathogenesis arises from dysregulated hyperproliferation and hyaline matrix deposition in dermal fibroblasts, manifesting transgressive expansion beyond initial wound boundaries through invasive tissue remodeling (Fong et al., 2014). Although research has advanced, the exact etiology of keloid is still unknown. Existing evidence associates keloid formation with multiple factors, such as growth factors, apoptosis - related genes, the immune microenvironment, and inflammation. However, despite multiple treatment options, high recurrence rates remain a significant challenge for both surgical and conservative approaches (Brown et al., 2008), often resulting in dissatisfaction with cosmetic outcomes, increased treatment costs, and reduced quality of life. These challenges highlight the need for novel molecular markers to prevent keloid progression and improve prognosis.

This study sought to elucidate molecular biomarkers implicated in keloid formation and progression, as well as potential therapeutic targets. Using an integrated dataset of 28 keloid and 23 control samples, we identified 679 DEGs. KEGG and GO enrichment analyses of these DEGs revealed primary enrichment in biological processes such as system development, suppression of cellular processes, and enhancement of cellular metabolic processes; cellular components including the cytoplasm; and molecular functions like catalytic activity. KEGG analysis identified significant pathway enrichment in lysosomes, cell cycle, ubiquitin-mediated protein degradation, and protein processing in the endoplasmic reticulum.

Through WGCNA and PPI network analyses, we identified two key genes, CDK7 and DDB2, associated with keloid. ROC curve analysis demonstrated that in validation datasets GSE121618 and GSE145725, the diagnostic accuracy of these two genes was high, with an AUC >0.9. Further validation via qRT-PCR, western blotting, and immunofluorescence confirmed significantly elevated expression of CDK7 and DDB2 in keloid specimens relative to normal specimens. These findings suggest that CDK7 and DDB2 may serve as valuable diagnostic markers for keloid.

The cyclin-dependent kinase (CDK) superfamily comprises evolutionarily conserved serine/threonine protein kinases functionally dedicated to orchestrating critical checkpoints in mitotic progression and transcriptional machinery activation. CDK7 emerges as a central regulatory component within this enzymatic hierarchy, exhibiting dual functionality in coordinating both cell cycle phase transitions and RNA polymerase II-mediated transcriptional initiation (Stephin J et al., 2021; Markéta et al., 2023). Studies show that reducing CDK7 activity in cancer cells, either genetically or pharmacologically, decreases cell proliferation, making CDK7 a promising therapeutic target in oncology (Zhang et al., 2024; Han et al., 2025). Given the pathological classification of keloid as benign fibroproliferative neoplasms, CDK7 might also function as a possible treatment target for keloid therapy.

Damage-specific DNA binding protein 2 (DDB2) functions as a UV-responsive genomic surveillance factor, specializing in detecting photolesions induced by ultraviolet radiation. Functioning as a genomic surveillance factor, the 48 kDa polypeptide encoded by the DDB2 gene orchestrates the detection of UV-induced DNA photolesions and initiates the mobilization of nucleotide excision repair (NER) complexes through phosphorylation-dependent signaling cascades (Scrima et al., 2008). Beyond its role in DNA repair, DDB2 acts as a multifunctional protein in cancer progression, serving as a modulator with dual functions that exhibit both tumor-promoting and tumor-suppressing activities (Roy et al., 2013). Experimental evidence demonstrates that DDB2 deficiency elevates oncogenic vulnerability in UV-irradiated murine models, with knockout murine systems exhibiting significantly enhanced spontaneous tumorigenesis rates (Itoh et al., 2004; Yoon et al., 2005). Research by Itoh T, Roy N, and Yang Z, among others, suggests a protective role for DDB2 in preventing skin tumor formation (Itoh et al., 2004; Roy et al., 2010; Yang et al., 2021). The elevated expression of DDB2, a key DNA damage recognition protein, in keloid—a benign fibroproliferative lesion—raises an intriguing question. It may suggest the presence of sustained, low-level genotoxic stress within the keloid microenvironment, potentially driven by chronic inflammation or oxidative stress. Alternatively, DDB2 might play a non-canonical role in keloid fibroblasts, independent of nucleotide excision repair, such as participating in the regulation of gene transcription or modulating key signaling pathways like TGF-β.

The TGF-β/Smad signaling pathway is a central pathway in the pathogenesis of keloid. It drives scar formation and progression by continuously activating keloid fibroblasts, leading to excessive deposition of extracellular matrix (ECM) (Hyun Jee and Yeong Ho, 2024). Previous studies have shown that the CDK7 inhibitor THZ1 blocks the activation of the TGFβ/Smad signaling pathway by inhibiting the phosphorylation of Smad2 (Jie et al., 2019). CDK7 is a key kinase in transcriptional regulation and may indirectly regulate the phosphorylation state of Smad2 by affecting the transcription of certain kinases or phosphatases. However, this effect has not been verified in keloid cell models. Future studies should further confirm this by treating primary keloid fibroblasts with THZ1 and detecting Smad2 C-terminal phosphorylation levels. In addition, Gaigai W et al. found that DDB2 is an upstream positive regulator of the TGF-β pathway, stabilizing p-SMAD2 by inhibiting NEDD4L, thereby enhancing the tumor suppression function of TGF-β (Gaigai et al., 2024). Currently, research has not clearly found whether CDK7 and DDB2 can affect the pathogenesis of keloid through the TGF-β/Smad signaling pathway. Although the specific molecular mechanism still needs further research, this provides a new perspective for understanding the role of CDK7 and DDB2 in the pathogenesis of keloid.

In summary, we identified two novel keloid-associated genes, CDK7 and DDB2, through bioinformatics analysis. We validated their functions and diagnostic efficacy through expression profiling, enrichment analysis, and ROC analysis. Further validation using qRT-PCR, western blotting, and immunofluorescence confirmed the reliability of our data analysis. Furthermore, we identified candidate therapeutic agents and regulatory transcription factors associated with these genes, which could represent novel therapeutic targets for keloid treatment. Based on the high expression characteristics of CDK7 and DDB2, targeted therapeutic strategies can be developed in the future: For example, combining CDK7 inhibitors (THZ1, alvocidib) with local drug delivery systems (nanogels) for precise local injection into keloid lesions to reduce systemic toxicity. Additionally, DDB2-specific small interfering RNA (siDDB2) can be designed and transfected into keloid fibroblasts via liposomal carriers to inhibit excessive collagen synthesis, providing a new non-surgical treatment option for keloids.

However, our study has several limitations. First, despite using four datasets, a potential limitation is the relatively small sample size of both the integrated GEO dataset and wet-lab validation. Future studies should validate CDK7/DDB2 expression in a multicenter cohort (n > 50 per group) to confirm their diagnostic utility across diverse patient populations. Second, the specific roles of CDK7 and DDB2 in keloid pathogenesis are not fully understood and require molecular biology experiments and rigorously designed multicenter studies for validation. Lastly, although CDK7 and DDB2 are recognized in oncology research, keloids are not classified as true tumors, necessitating further experimentation to substantiate our findings.

## 5 Conclusion

Integrated bioinformatics and basic experimental approaches were used in this study to detect and confirm two biomarkers, CDK7 and DDB2, associated with keloid. These markers hold potential as future targets for diagnosing and managing inflammation in keloid.

## Data Availability

The datasets presented in this study can be found in online repositories. The names of the repository/repositories and accession number(s) can be found in the article/supplementary material.
